# Maxillary Protrusion with Severe Overjet Treated by Maxillary Anterior Alveolar Osteotomy

**DOI:** 10.1155/2024/3850765

**Published:** 2024-03-09

**Authors:** Daisuke Tomita, Shugo Haga, Fumi Tanzawa, Taira Miyazawa, Haruhisa Nakano, Koutaro Maki

**Affiliations:** ^1^Department of Orthodontics, Showa University, 2-1-1 Kitasenzoku, Otta-ku, Tokyo 145-8515, Japan; ^2^Department of Orthodontics, School of Dentistry, Showa University, 2-1-1 Kitasenzoku, Otta-ku, Tokyo 145-8515, Japan

## Abstract

In addition to affecting oral functions such as temporomandibular joint function, mastication, and speech, malocclusion caused by skeletal maxillary prognathism also entails sociopsychological implications. Surgical orthodontic treatment to improve occlusion and oral function and to correct esthetic disharmony is important to improve patients' quality of life. We report the case of a 32-year-old woman who visited our hospital with a chief complaint of proclined upper front teeth. Clinical examination revealed maxillary overgrowth and severe labial inclination of the maxillary incisors with palatal gingival recession. The incisal protrusion was corrected with a maxillary anterior alveolar osteotomy—a surgical orthodontic method that could improve the overbite without causing excessive lingual inclination, while also minimizing orthodontic movement of the maxillary anterior teeth. This treatment is generally indicated in cases of maxillary prognathism with a relatively stable occlusal relationship in the molar region. As a result of the treatment, the patient's chief complaint improved and a long-term functional occlusion was achieved. This paper outlines the pre- and posttreatment skeletal and occlusal changes.

## 1. Introduction

Increased protrusion of the maxillary anterior teeth due to a prognathic maxilla is a common orthodontic problem. This in turn compromises the facial esthetics, especially the soft tissue profile [1]. Moreover, the increased overjet might contribute to trauma and impingement of the palatal tissues, resulting in damage to the periodontium lingual to the maxillary incisors [2]. Individuals with increased maxillary prognathism can be treated successfully by orthodontic treatment involving retraction of the maxillary anterior teeth if the discrepancy is not severe [3, 4]. However, in more severe cases, it is often difficult to improve the overjet by moving the teeth alone, and prolonged treatment, including root resorption and gingival recession, also presents additional risks [5, 6]. It is therefore important to consider combined surgical and orthodontic treatment in cases with significant maxillary prognathism that cannot be camouflaged by orthodontic tooth movement alone. One of the most common treatment modalities in patients with skeletal maxillary prognathism is anterior maxillary alveolar osteotomy or Wassmund's method. The maxillary anterior alveolar osteotomy technique is primarily employed to posteriorly reposition the anterior dentoalveolar segment through a limited vestibular approach. It is usually indicated in patients with excessive dentoalveolar protrusion and relatively stable favorable molar occlusion. This technique has many advantages, including that sever the sagittal skeletal discrepancy, while the axial inclination and vertical position of the anterior teeth are controlled [7]. Furthermore, minimizing excessive and prolonged tooth movement reduces the risk of root resorption and gingival recession [8]. In this article, we report a patient with a skeletal Class II abnormality resulting from maxillary prognathism who was treated by maxillary anterior alveolar osteotomy.

## 2. Diagnosis and Etiology

**Figure 1:**
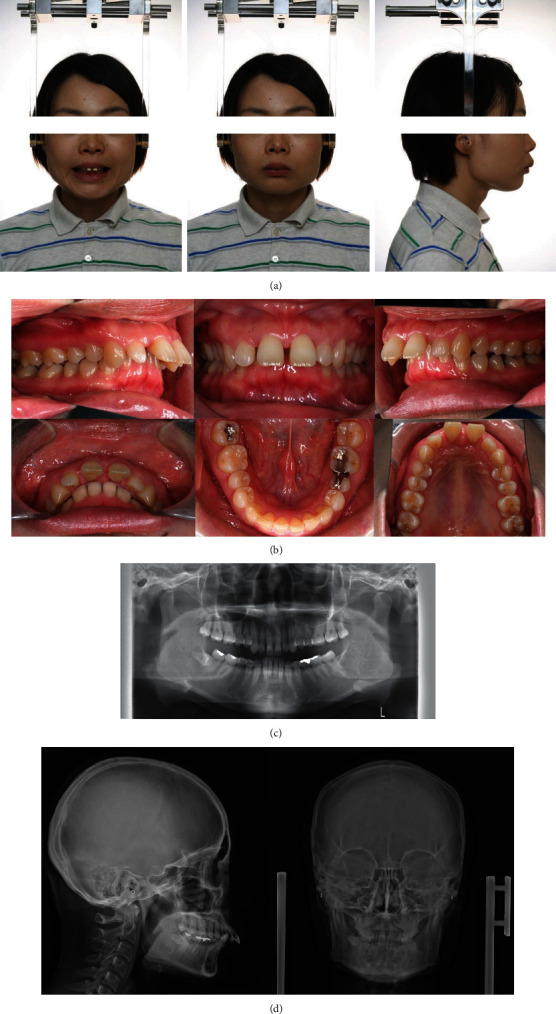
Pretreatment records. (a) Extraoral photographs. (b) Intraoral photographs. (c) Orthopantomograms. (d) Posteroanterior and lateral cephalograms.

A 32-year-old woman visited the Orthodontic Department for blinded review with a chief complaint of forwardly placed upper front teeth. The patient had no significant medical history. The frontal appearance of the patient showed a euryprosopic asymmetric face due to mandibular shift to the right, incompetent lips, and a reduced lower facial height. Her profile showed increased facial convexity with a protruded upper lip. Intraoral examination revealed an Angle Class II, division 1 malocclusion with an overjet of +13.5 mm, overbite of +9.5 mm, and a curve of Spee of 3.0 mm. The maxillary and mandibular midlines were coincident with the midsagittal plane. Lingually, the clinical attachment level of the gingival tissue of the maxillary central incisors was reduced due to traumatic contact with the mandibular central incisors on both sides, exposing 3.0 mm of the roots (Figures 1(a) and 1(b)).

Panoramic and temporomandibular joint view radiographs showed that the patient had bilateral deformities of the condyles (Figures 1(c) and 1(d)). Lateral cephalometric analysis revealed skeletal Class II due to maxillary prognathism with a reduced mandibular plane angle and severely proclined upper and lower incisors. Soft tissue analysis revealed protruded upper and lower lips in relation to the E-line (Table 1). Posteroanterior cephalometric analysis showed that the mandible was deviated to the right as a result of decreased ramus height on the right side.

## 3. Treatment Plan

The treatment objectives were to reduce the maxillary projection, achieve ideal overjet and overbite while maintaining a Class II molar relationship, correct the lip incompetency and level of the curve of Spee, and achieve a mutually protected functional occlusion, with stable and simultaneous occlusal contacts of all teeth in centric and eccentric contacts.

**Figure 2:**
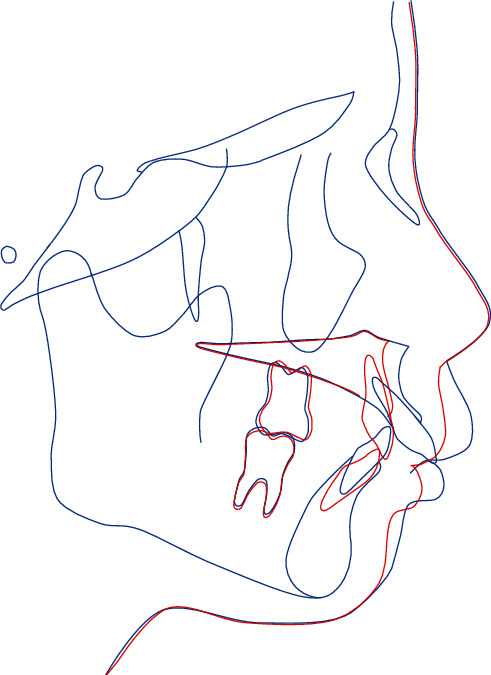
Surgical treatment objective: pretreatment (blue line) and posttreatment (red line).

However, significant gingival recession was observed in the cervical region on the lingual side of the maxillary anterior teeth. Therefore, to reduce the amount of movement required by orthodontic treatment, a combined approach of orthodontic treatment and surgical correction was planned. In the maxillary arch, it was planned to extract the first premolars, followed by a maxillary anterior alveolar osteotomy to position the anterior maxilla posteriorly by 5 mm. In the mandibular arch, we planned to level the curve of Spee and retract the lower anterior teeth (Figure 2). The patient provided written informed consent for the procedure.

## 4. Treatment Progress

### 4.1. Phase I: Presurgical Orthodontics

**Figure 3:**
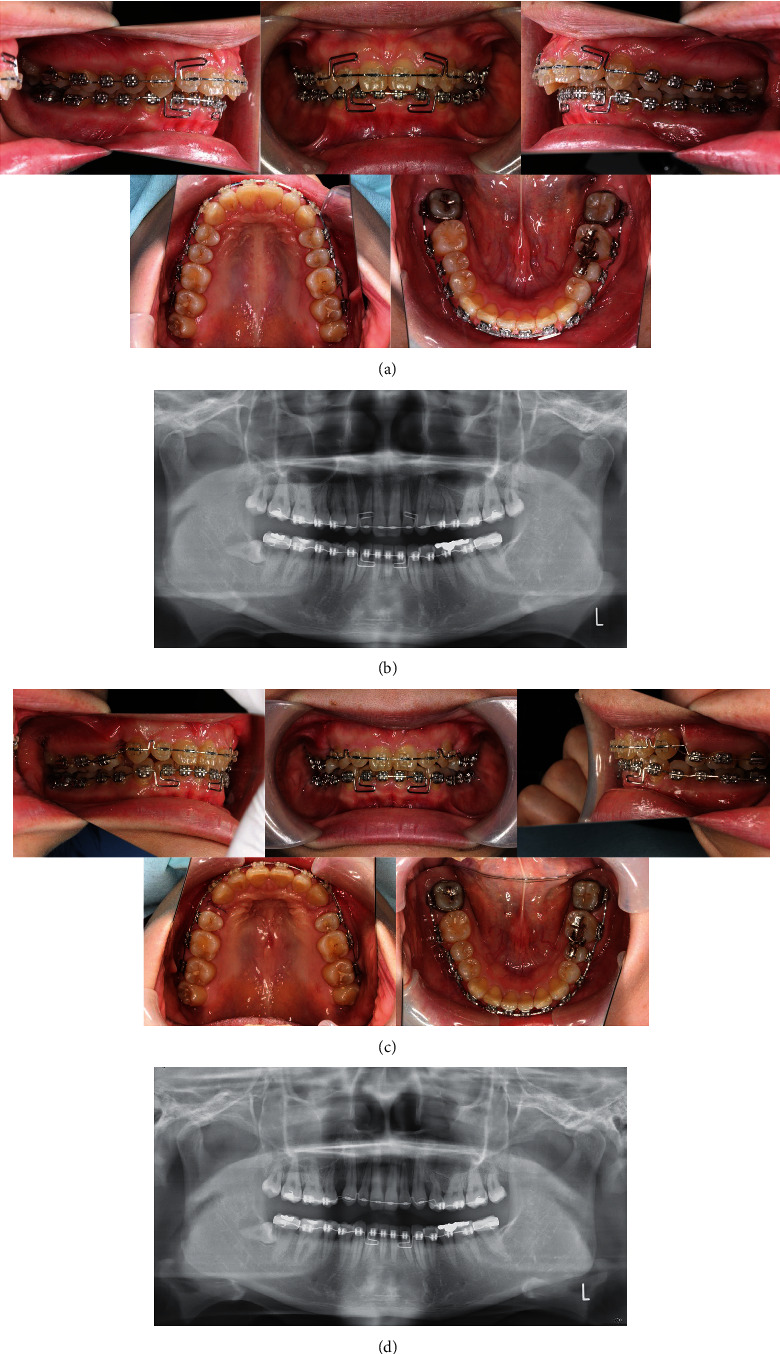
Presurgical and postsurgical records. (a) Presurgical intraoral photographs. (b) Postsurgical intraoral photographs.

The presurgical orthodontic phase was started by bonding 0.018^″^ standard edgewise brackets to all teeth. The upper and lower dentition were leveled with continuous 0.014^″^ and 0.016^″^ nickel-titanium wires and 0.016^″^ stainless steel archwires. The maxillary arch was then detailed using 0.016 × 0.022^″^ and 0.017 × 0.022^″^ stainless steel wires. The deep curve of Spee was corrected with a 0.016 × 0.022^″^ stainless steel curved wire with an L-loop. Correction of the curve of Spee revealed a space in the anterior region distal to the lower incisors, which was closed by activating the L-loop (Figure 3(a)). Before completing the presurgical orthodontic phase, a study model was obtained to evaluate the arch coordination, occlusion, and overjet. The final amount of surgical movement after 20 months of presurgical orthodontics was determined by lateral and posteroanterior cephalograms and cone-beam computed tomography imaging. Mock surgery was performed on models mounted on a semiadjustable articulator involving extraction of the maxillary first premolars and posterior positioning of the maxillary anterior segment by 5.0 mm, as planned. Positioning splints were fabricated, and intermaxillary fixation hooks were attached between the brackets with continuous 0.016^″^ × 0.016^″^ stainless steel archwires for fixation. Presurgical orthodontic treatment duration was 18 months.

### 4.2. Phase II: Surgical Procedure

Under general anesthesia, the maxillary first premolars were extracted, followed by maxillary anterior alveolar osteotomy to posteriorly position the premaxillary segment, based on the Wassmund method. The anterior maxillary segment was moved backwards by 5.0 mm and fixed with an absorbable miniplate. A 0.016^″^ × 0.016^″^ stainless steel wire was inserted intraoperatively and passively continuously ligated to prevent space opening at the extraction site after surgery. After surgery, the wound was checked daily for 1 week for any surgical complications. The splint was kept in place for 4 weeks, and the patient was placed on a liquid diet.

### 4.3. Phase III: Postsurgical Orthodontics

After surgery, 0.016^″^ × 0.022^″^ stainless steel wire with Class II elastics was used to close the minor spaces distal to the canines and to level the canines and premolars (Figure 3(c)). A study model was then obtained to evaluate the occlusion and the need for further finishing and detailing. In particular, the brackets on the maxillary canines on both sides were repositioned because the roots were previously tipped mesially to avoid damage during the osteotomy.

**Figure 4:**
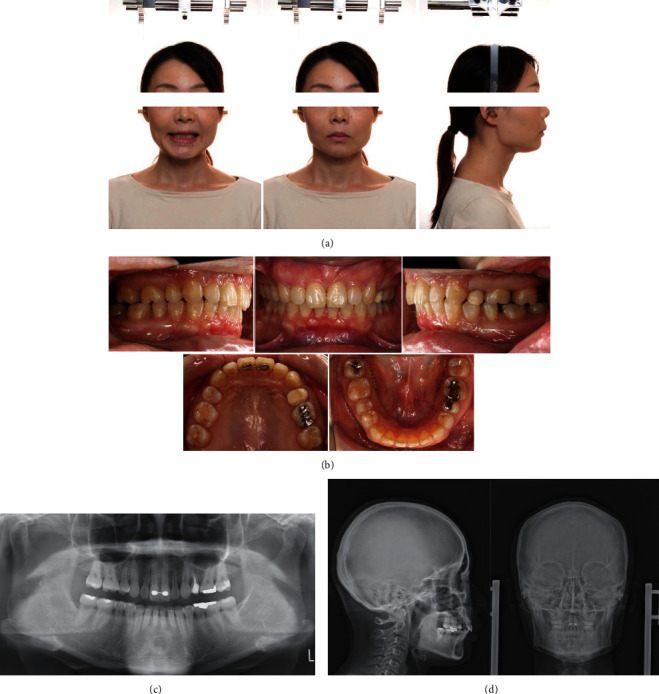
Posttreatment records. (a) Extraoral photographs. (b) Intraoral photographs. (c) Orthopantomograms. (d) Posteroanterior and lateral cephalograms.

**Figure 5:**
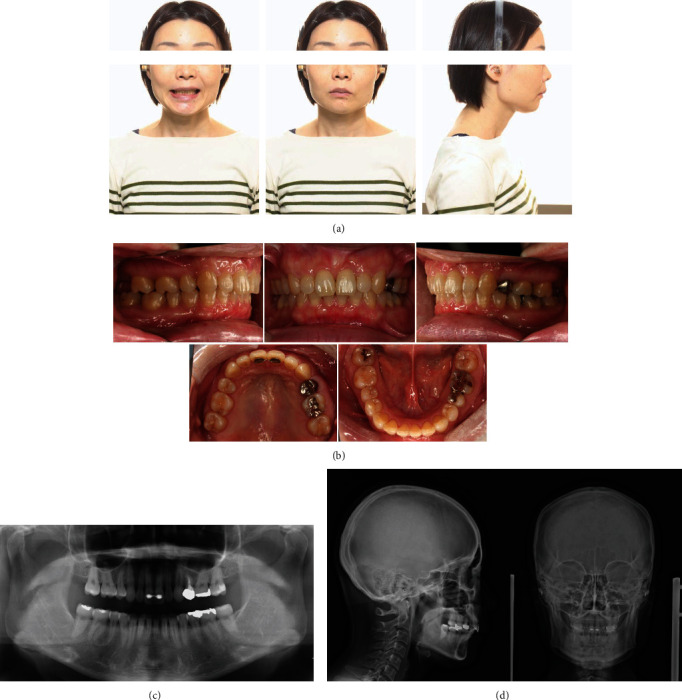
Two-year posttreatment records. (a) Extraoral photographs. (b) Intraoral photographs. (c) Orthopantomograms. (d) Posteroanterior and lateral cephalograms.

After completion of the finishing and detailing stage, the appliance was debonded. Postsurgical orthodontic treatment duration was 31 months. The Hawley retainers were used for the upper and lower arches, in addition to a fixed retainer bonded to the maxillary central incisors (Figures 4(a)–4(d)). The overall active treatment duration was 49 months. Full set of records was obtained 2-year posttreatment to evaluate the stability of the treatment results (Figures 5(a)–5(d)).

## 5. Treatment Results

**Figure 6:**
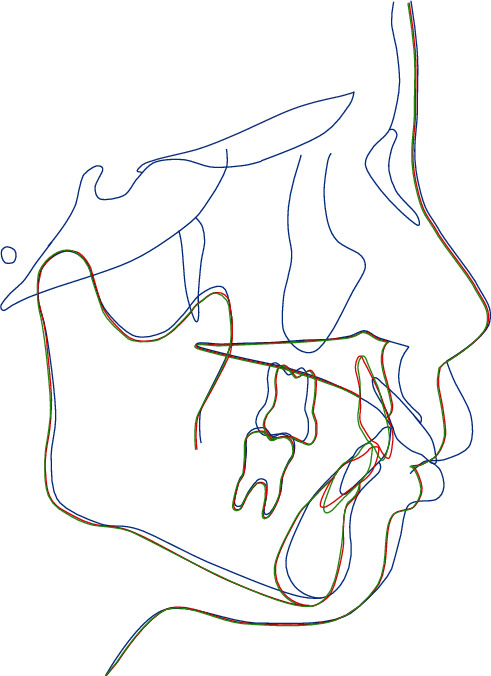
Superimposed cephalometric tracing at pretreatment (blue line), posttreatment (red line), and 2-year posttreatment (green line).

There was an improvement in facial esthetics, with decreased facial convexity and improved lip competency. Intraoral examination showed that the previously observed deep bite condition, where the lower anterior teeth contacted the lingual gingiva of the upper anterior teeth during the initial examination, had improved, with an overjet and overbite of 2.5 mm, indicating improved vertical and horizontal relationships of the anterior teeth. The molar and canine relationships were Angle Class II (Figures 4(a) and 4(b)). Lateral cephalometric analyses at the time of the initial examination, after treatment, and after 2 years of retention are shown in Table 1. Skeletally, the SNA angle improved from 82.6° to 75.9°, the SNB angle from 73.1° to 70.1°, and the ANB angle from 9.4 to 5.8. Moreover, the mandibular plane angle changed from 18.9° to 20.9°. Dentally, the U1 to FH plane angle changed from 127.1° to 107.2°, and the L1-mandibular plane angle changed from 108.0° to 115.0° (Figures 4(c) and 4(d)). The tracings of the pretreatment, posttreatment, and 2-year posttreatment cephalograms showed that the results were stable (Figure 6). There was no change in gingival recession of the maxillary bilateral central incisors compared with the preoperative period and no significant change in the results of the pocket examination (Figures 4(b) and 5(b)).

## 6. Discussion

In the present case, we used maxillary anterior alveolar osteotomy (Wassmund's method) on the maxillary first premolars to posteriorly position the anterior dentoalveolar segment. The usual indications for this procedure are excessive inclination of the anterior teeth and excessive vertical or sagittal development of the maxillary alveolar process, with a relatively good buccal occlusal relationship [9]. This method has several advantages, including dramatic improvement of skeletal and dental discrepancies, superior postoperative stability, and reduction of the required tooth movement and hence treatment time [7]. Although root resorption associated with orthodontic tooth movement is difficult to predict, previous reports indicated that the amount of tooth movement and treatment time were risk factors for root resorption [10]. Furthermore, the maxillary anterior teeth are more susceptible to root resorption associated with orthodontic tooth movement [11].

**Figure 7:**
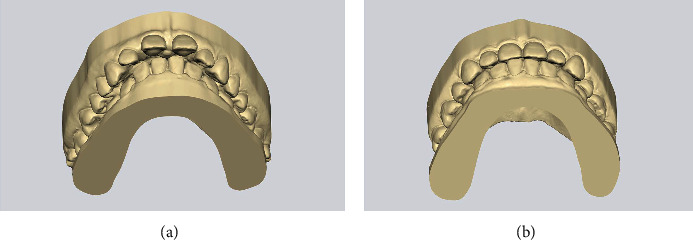
Pre- and posttreatment records showing the changes in anterior occlusion. (a) Pretreatment record. (b) Posttreatment records.

In clinical practice, it is necessary to pay attention to the root tip of the teeth adjacent to the osteotomy site before starting presurgical orthodontic treatment. The presurgical orthodontic plan includes mechanics to tip the roots of the teeth adjacent to the osteotomy site away from the cuts, especially the canines, which have the longest roots and are the most susceptible to injury. Injury to the adjacent teeth might lead to loss of vitality and periodontal damage, with subsequent gingival recession. Therefore, in the presurgical orthodontic treatment, we moved the canine root mesially to avoid the osteotomy site. In addition, harmonizing the anterior and posterior dental arch widths should be considered during presurgical orthodontic and surgical procedures. The bite was adjusted to a Class II molar relationship from the first premolar to the second molar during the presurgical orthodontic treatment. For the anterior teeth, the arrangement was made using study models to achieve an appropriate overjet, overbite, and intercanine width (Figures 7(a) and 7(b)). During surgery, maximum fixation with absorbable miniplates and continuous ligation of the archwire were essential to prevent opening of the extraction space and improve postoperative stability.

The current patient presented with skeletal maxillary prognathism and significant protrusion of the maxillary anterior teeth, together with an Angle Class II molar relationship and anterior deep overbite. These criteria were clear indications for anterior maxillary alveolar osteotomy. During this procedure, posterior movement and clockwise rotation of the anterior dentoalveolar segment allowed correction of the proclined maxillary incisors, increased overjet, and increased deep overbite. Initial examination of the patient showed severe gingival recession lingual to the maxillary central incisors [8]. Previous studies indicated that gingival recession is a multifactorial condition, with abnormal inclination of the teeth and traumatic occlusion among the contributing factors. The excessive inclination of the maxillary incisors in the current case might have been associated with severe bone loss caused by the abnormal inclination and position of the teeth. In addition, the absence of an incisal stop anteriorly due to the increased overjet led to an impinging deep overbite, which was in turn detrimental to the periodontal tissues and supporting alveolar bone lingual to the maxillary incisors [12]. Both these factors might have contributed to the gingival recession seen in this patient. In addition, the plaque adhered to the retracted area and the patient suffered from mild gingivitis, which may have led to a more severe gingival recession, creating a vicious cycle. If the occlusal interference was not resolved, the tooth movement and gingival recession would worsen, and immediate orthodontic treatment was therefore needed to correct the problem. The final treatment results showed a 10.0 mm reduction in the overjet, reduction of facial convexity, and improved lip competency. With regard to the canines, the results showed slight mesial movement of the buccal segment to produce a Class II relationship. This indicated that there might have been some anchorage loss, which could be avoided by reinforcing the anchorage with an appliance, such as a temporary anchorage device. The presurgical orthodontic treatment period was 18 months, and the postsurgical orthodontic period extended to 31 months, which resulted in a prolonged postsurgical phase. However, it is possible that this prolongation could have been prevented by strengthening the anchorage during treatment. Examination of the patient 2 years after the procedure showed no significant change in the treatment results, indicating the stability of the procedure.

## 7. Summary and Conclusions

This patient had dramatic skeletal, dental, and occlusal improvements following the use of the maxillary anterior alveolar osteotomy technique combined with orthodontic treatment. Moreover, there was a notable improvement of her gingival recession attributed to the diagnosed malocclusion. At her 2-year follow-up, the patient had achieved esthetic, functional, and stable outcomes. The patient was also satisfied with the results and reported improved self-esteem. This technique is considered an important factor controlling root resorption associated with orthodontic tooth movement.

## Figures and Tables

**Table 1:** Lateral cephalometric measurements.

Measurement	Normal	Pretreatment	Retention	After retention for 2.0 years
*Skeletal pattern*				
SNA (°)	82.3	82.6	75.9	75.7
SNB (°)	78.9	73.2	70.1	70.5
ANB (°)	3.4	9.4	5.8	5.9
*y*-axis (°)	65.4	62.8	65.3	65.7
SN.GoGn (°)	34.9	25.1	32.7	32.9
FMA (°)	28.8	18.9	20.9	20.3

*Dental pattern*				
1.NA (°)	22.1	33.4	19.4	13.6
1-NA (mm)	5.4	7.1	0.5	0.1
1.NB (°)	29.5	31.2	37.9	30.1
1-NB (mm)	7.4	8.3	6.5	6.7
IMPA (°)	96.3	108	115	107.1

## Data Availability

All data related to the presented case are included in this published article.
